# Investigations at the Product, Macromolecular, and Molecular Level of the Physical and Chemical Properties of a γ-Irradiated Multilayer EVA/EVOH/EVA Film: Comprehensive Analysis and Mechanistic Insights

**DOI:** 10.3390/polym13162671

**Published:** 2021-08-10

**Authors:** Fanny Gaston, Nathalie Dupuy, Nina Girard-Perier, Sylvain R. A. Marque, Samuel Dorey

**Affiliations:** 1 Sartorius Stedim FMT S.A.S, Z.I. Les Paluds, Avenue de Jouques CS91051, CEDEX, 13781 Aubagne, France; nina.girard@sartorius.com; 2 Aix Marseille Univ, Avignon Université, CNRS, IRD, IMBE, 13013 Marseille, France; 3 Aix Marseille Univ, CNRS, ICR, Case 551, 13397 Marseille, France

**Keywords:** γ-irradiation impact, Ethylene Vinyl Acetate, polymers, multilayer film, pharmaceutical and biotechnological single-use systems

## Abstract

Chemically and biologically safe storage of solutions for medical uses is a daily concern for industry since decades and it appeared even more dramatic during the last two years of pandemia. Biological safety is readily reached by sterilization using γ-irradiation process. However, such a type of irradiation induces the degradation and the release of chemicals able to spoil the biological solutions. Surprisingly, there are no investigations on multi-layer films combining multi-technique and multi-method approaches to unveil the events occurring during γ-irradiation. Furthermore, our investigations are focuses on properties/events occurring at product, macromolecular, and molecular levels.

## 1. Introduction

Single-use systems are more and more frequently used in the biopharmaceutical and biotechnological industries. Plastic bags provide a single-use alternative to traditional containers made of glass, stainless steel, and rigid plastic, thus, avoiding difficult cleaning and potential cross-contamination between several successive uses. These single-use bioprocessing bags are flexible containers manufactured from multilayer films, like polyethylene (PE), ethylene vinyl acetate (EVA), and ethylene vinyl alcohol (EVOH), and are designed for the preparation, storage, and transport of biopharmaceutical solutions, intermediates, and final bulk products [1,2,3]. It is easy to imagine how important it is, in these COVID-19 times, that the end-users should be provided with efficiently sterilized materials, and that the integrity of materials (e.g., permeation, thermal, and mechanical properties) and the quality of the stored solutions (e.g., acidity, oxidation, pollution) should not be altered by the sterilization by γ-irradiation. Indeed, any deterioration of these properties would lead to these solutions (often several cubics of them) being disposed of, which would generate delays in the delivery of raw (or elaborate) material useful to fight epidemia.

Thus, according to the regulation, the conventional γ-irradiation dose used in biopharmaceutical industries is >25 kGy [4]. The major advantage of this mode of sterilization is the high penetration capability [5,6] of the irradiation, reaching all parts of the single-use plastic systems and affording the highest level of sterilization, while keeping most of the material properties unaffected. Nevertheless, issues concerning unwanted acidity and oxidation events have been reported by end-users of γ-sterilized EVA/EVOH/EVA multi-layer films (vide infra). Surprisingly, only very few studies [7] involving a multi-technique approach have investigated the effects of γ-irradiation on multi-layer films. By contrast, such effects have been intensively studied on mono-layer films such as EVOH, PE, or EVA films [8,9,10,11,12,13,14,15,16,17,18,19,20].

Thus, taking into account the lack of multi-technique approach, we developed a multi-level approach for the investigation of EVA/EVOH/EVA multi-layer films: (i) analysis of the properties at the product level, focusing on those material properties of the utmost importance for the end-users, e.g., the integrity of the materials and the quality of the stored solutions; (ii) analysis at the macromolecular level to get insights into the chemical changes leading to deterioration of material integrity; and (iii) analysis at the molecular level to understand all chemical processes leading to the observations done in (i) and (ii).

Such a multi-level and multi-technique approach generates a huge amount of data (e.g., thousands FTIR spectra, hundreds HPLC traces). Thus, the analysis of these data is performed using chemometrics methods such as Principal Component Analysis PCA and AComDim and an experimental design approach. It provides invaluable insight into the interaction between events occurring at the molecular level and the properties of the material intended for a daily use. In addition, it is a powerful tool that can help in proposing solutions for the sometimes antagonistic requests of end-users. Hence, during the last decade, we have focused our investigations on EVA/EVOH/EVA film, this material being selected for its flexibility and barrier properties [21,22]: EVA is a barrier to water and EVOH is a barrier to carbon dioxide CO_2_ and dioxygen O_2_. Thus, this article proposes a comprehensive discussion, dealing with molecular mechanisms as well as material properties, reported in all articles published by our groups during the last decade. Hence, it will provide to the reader a general overview of the complexity of the chemistry occurring in such materials and of how powerful our systemic approach is.

Importantly, our investigations were performed on ready-to-use materials prepared in the factory and the irradiations were performed in sterilization companies in the industrial conditions. This approach has a strong limitation due to the non-controlled environment, which was circumvented by taking care on repeatability and reproducibility on the experiments. On the other hand, this approach has a tremendous advantage, which is to work in real condition with the company preparing the materials and the companies performing the irradiation. Then, the results obtained from the multi-level and multi-level technique approach are straightforwardly applicable by the end-users.

## 2. Generalities

**Figure 1:**
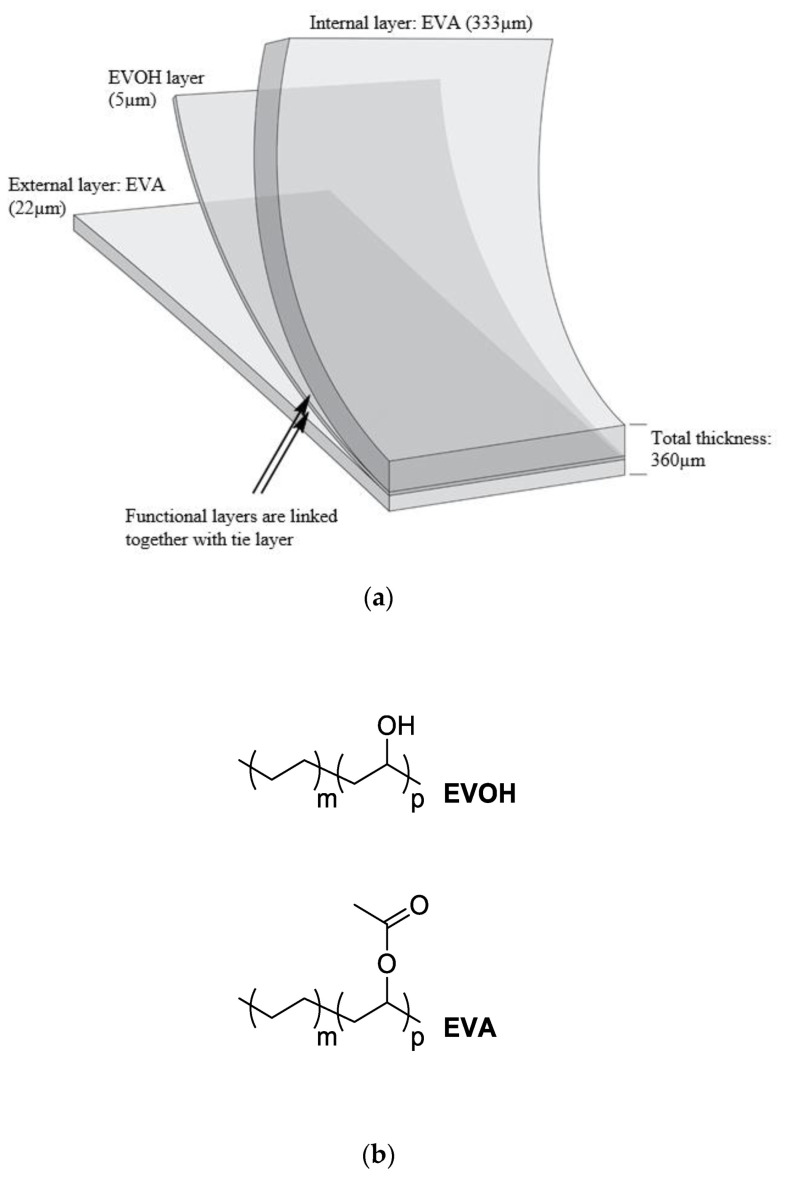
Structure of EVA multi-layer film (**a**) and structure of polymers (**b**). Reproduced from [34] with the permission of European Journal of Pharmaceutical Sciences.

The thickness of the EVA/EVOH/EVA multilayer film investigated was about 360 µm (Figure 1). The different layers of the EVA-film contained additives for the stabilization of the film throughout the manufacturing process and throughout its shelf-life [23,24,25,26]. EVA/EVOH/EVA -film samples were packed and wrapped in specific packaging (PE) and irradiated at room temperature by means of a ^60^Co γ-source, affording doses at 30 (±1), 50 (±1), 115 (±2), and 270 (±5) kGy [27]. Higher doses than needed (<50 kGy for the sterilization of materials for commercial applications) were applied to magnify the modifications induced by γ-irradiation, considering that the modifications occurring at high doses also occur in lower proportions at lower doses. Importantly, results were repeatable and reproducible, although the irradiation procedure was rather an uncontrolled procedure.

This EVA/EVOH/EVA multi-layer film was investigated using several techniques: Fourier Transform InfraRed (FTIR) spectroscopy [28], Electron Paramagnetic Resonance (EPR) [29], colorimetry measurements [30], HPLC to measure the oxidation event [31] pH, conductivity, Total Organic Carbon (TOC), ion chromatography [32], X-ray Photoelectron Spectroscopy (XPS) [33], tensile tests, Differential Scanning Calorimetry (DSC), Oxygen Transmission Rate (OTR), and Water Vapor Transmission Rate (WVTR) [34]. All these techniques generated a large amount of data, which were analyzed using chemometrics methods such as Principal Component Analysis (PCA) [35] and ANOVA Common Dimensions (AComDim, Analysis of Variance in Common Dimensions) [36]. Moreover, a full design experiment approach was devised as a predictive tool to provide solutions for the requests of end-users [37,38].

**Scheme 1:**
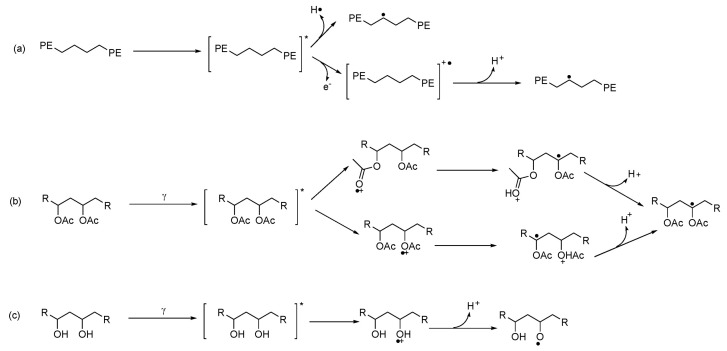
Generation of radicals from PE (**a**), PVA (**b**), and PVOH (**c**).

The main event occurring during sterilization of materials by γ-irradiation is the generation of radical cations (first generation radicals) yielding instantaneously second-generation radicals, which cause all the changes in material properties discussed hereafter. The EVA/EVOH/EVA multi-layer film is composed of three different types of polymer, affording the generation of several types of radicals for each type of polymer, i.e., PE (Scheme 1a), PolyVinyl Acetate (PVA) (Scheme 1b), and PolyVinyl Alcohol (PVOH) (Scheme 1c).

For the sake of simplicity, under γ-irradiation, only the loss of one electron from the lone pairs of O-atoms is assumed in EVA and EVOH polymers (loss of H• on the backbones affords radicals species which are discussed in Section 6). That assumption is done because the lone pairs of electrons request, in general, a low level of energy to be removed [39].

The fate of these radical species will rule the changes observed in the material properties, and analyses at the material, macromolecular, and molecular level will provide insights into the mechanisms underlying these changes.

## 3. Analysis at the Product Level

Several properties, i.e., gas and liquid permeation, mechanical stress resistance, yellowness of materials, acidity of stored solutions, and potential of oxidation, were investigated using several techniques such as colorimetry, titration, HPLC, permeation, mechanical stress measurement.

### 3.1. Colorimetry

After γ-irradiation, the color of the multi-layer EVA/EVOH/EVA film changes from colorless to yellowish and a strong smell of acetic acid is released [30]. This means that y-irradiation generates chromophore compounds, arising from the degradation of the additives package, which includes phenol and phosphite additives [23,24,25,26].

**Figure 2:**
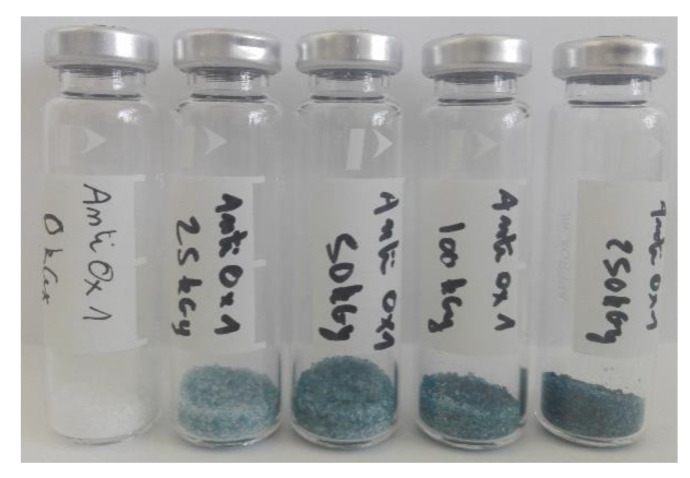
Non-irradiated and γ-irradiated 2,6-di-tert-butyl-p-cresol in solid phase (left to right: 0, 25, 50, 100, and 250 kGy).

**Figure 3:**
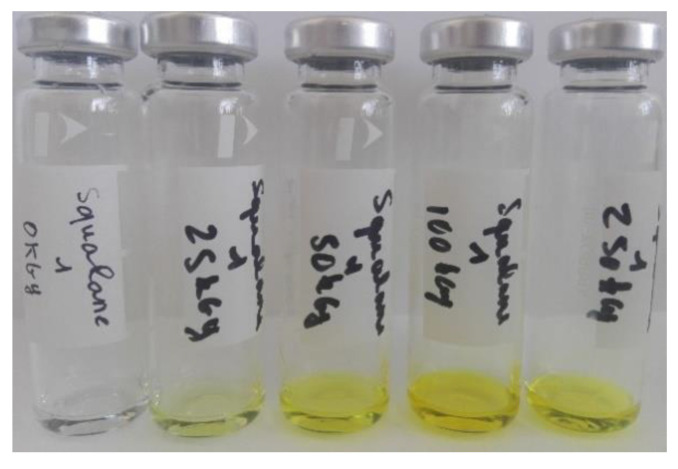
Non-irradiated and γ-irradiated 2,6-di-tert-butyl-p-cresol in polymer matrix simulating (left to right: 0, 25, 50, 100, and 250 kGy).

The additives seem to be responsible for the change in the color of the multi-layer EVA/EVOH/EVA-film, but yellowing is not directly observed on the individual additive in solid phase (powder state, dark blueish), as illustrated in Figure 2 with the 2,6-di-*tert*-butyl-*p*-cresol. When the additive is dissolved in squalene, hydrocarbon good at mimicking the polymer matrix [40]. γ-irradiation generates yellowing of the solution, as displayed in Figure 3. The package of additives is responsible for the yellowing of the multi-layer film upon γ-irradiation when they are contained in the polymer matrix.

### 3.2. Acidity

**Figure 4:**
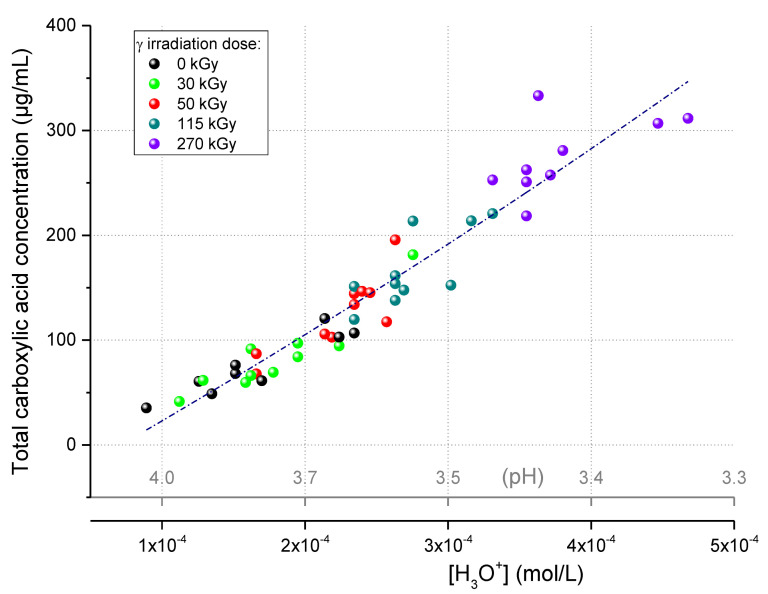
Total carboxylic acid content vs [H_3_O^+^] (the pH is given for information) at different γ-doses. The total carboxylic acid content is the sum of all the acid concentrations detected by ion chromatography. The dashed line shows the trend. Reproduced from [32] with the permission of European Journal of Pharmaceutical Sciences.

The quantity of carboxylic acids generated by γ-irradiation is detected by ionic chromatography, and the solution pH is determined by a conventional method [32]. It is observed that the total carboxylic acid concentration (Figure 4) increases with the irradiation dose, and that the solution pH is lower than 4 even for irradiation doses used for commercial products. The generation of such acids is confirmed by FTIR [28] and XPS [33] investigation.

**Scheme 2:**
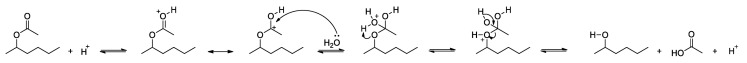
Hydrolysis of ethylene vinyl acetate. Reproduced from [28] with the permission of Polymer Degradation and Stability.

The smell of acetic acid already present before the γ-irradiation (confirmed by low pH, Figure 4) is ascribed to the hydrolysis of the vinyl acetate group (Scheme 2).

Ion chromatography indicates that formic and acetic acids are the main carboxylic acids generated, directly causing the changes in pH [32]. Their formation is discussed in the mechanism section. Some heavy acids are also detected and are ascribed to the oxidation of the PE copolymer [32]. However, their amount is so low that it has no significant impact on the changes in pH.

### 3.3. Permeability

**Figure 5:**
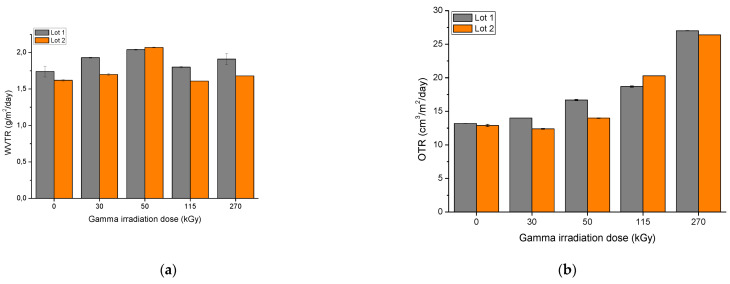
(**a**) WVTR and (**b**) OTR of EVA/EVOH/EVA multilayer film according to the γ-irradiation dose. Reproduced from [34] with the permission of Journal of Applied Polymer Science.

The EVA layer is the water barrier layer, its barrier property being mainly due to the polyethylene chains present in this copolymer. The EVA polymer is known to be a slightly porous material, with WVTR (water vapor transmission rate) about 2 g/m²/day. The measurement by WVTR of the water permeability properties of the EVA polymer [34] shows that they do not vary according to the γ-irradiation dose, as displayed in Figure 5a. Thus, the permeability properties observed for our materials are very close to those reported in the literature, meaning that, up to a dose of 270 kGy, cross-linking and scission events do not occur to an extent large enough so that the permeability properties of the material are modified.

The chemical environment of the EVOH layer, which is embedded between the EVA layers, is changed after γ-irradiation. The measurement by OTR (oxygen transmission rate) of the oxygen permeability properties of the EVA/EVOH/EVA film [34] highlights these changes in the chemical environment after γ-irradiation. The EVOH layer is more permeable to O_2_ after γ-irradiation, especially above 115 kGy, as highlighted in Figure 5b. This permeability is probably also due to the increase in the number of micro-channels and/or micro-voids in the EVOH layer after γ-irradiation. They help in the migration of O_2_ through this layer. The degradation of the EVOH layer is confirmed by DSC measurements (vide infra).

### 3.4. Mechanical Test

**Figure 6:**
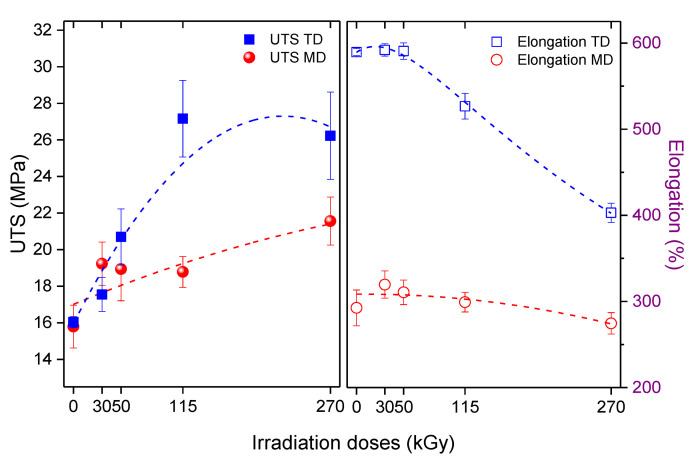
(**Left**) Ultimate Tensile Strength (UTS) for TD (blue solid squares) and MD (red solid circles), and (**right**) elongation for TD (blue open squares) and MD (red open circles) as a function of the γ-irradiation dose. Dashed fitting curves are prearranged. Reproduced from [34] with the permission of Journal of Applied Polymer Science.

The mechanical tests [34] performed along the machine direction (MD) and the transverse direction (TD) of the EVA/EVOH/EVA multi-layer film reveal an increase in the ultimate tensile strength (UTS) and a decrease in the elongation at break according to the γ-irradiation dose (Figure 6). For γ-doses larger than 115–270 kGy, the EVA/EVOH/EVA multi-layer film becomes less flexible. This increase in stiffness is surely due to cross-linking events occurring in the EVA layer polymers chains [41]. However, the doses used for commercial applications are not high enough to reach these mechanical conditions. Indeed, for EVA/EVOH/EVA films, the elongation is never higher than the elastic elongation.

### 3.5. Oxidation

**Figure 7:**
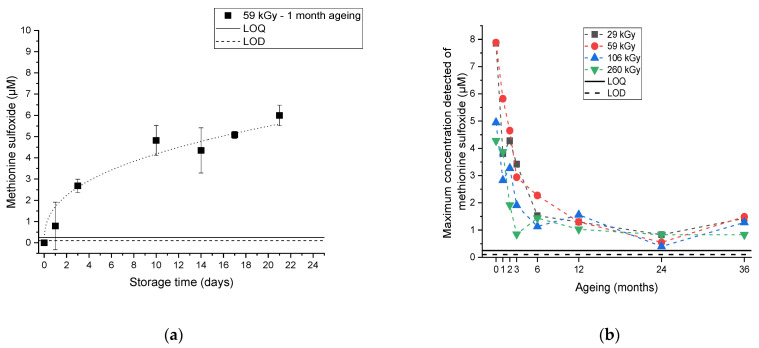
(**a**) Concentration in methionine sulfoxide vs storage time at room temperature for one-month aged bag irradiated at 59 kGy containing a solution of water (methionine = 50µM). (**b**) Plots of maximum of concentration in methionine sulfoxideF after 21 days of storage vs ageing of bag after irradiation at various doses. LOQ for limit of quantification and LOD for limit of detection. Reproduced from [42] with the permission of Polymers.

**Scheme 3:**
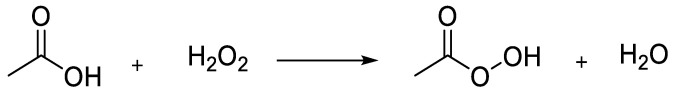
Formation of peracetic acid.

**Scheme 4:**
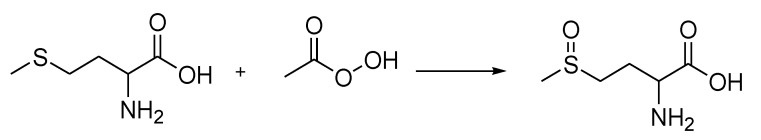
Formation of methionine sulfoxide.

Because of small amounts according to the polymers amount and of the absence of featuring signals, peroxides such as peracetic acid or hydrogen peroxide are unlikely to be detected by spectroscopic methods. By contrast, amino acids such as cysteine or methionine are often used to probe the presence of oxidant in biological solutions. Therefore, we adapted a procedure based on the oxidation of methionine to investigate the presence of oxidants such as peroxide, peracids, and hydrogen peroxide [42]. Importantly, it is well known that hydrogen peroxide reacts with acetic acid to generate peracid in situ (Scheme 3) [42,43] affording the methionine sulfoxide derivative (Scheme 4). Thus, with oxidation models using methionine, and taking into account a lengthy experience (up to 21 days for a one-month aged bag, Figure 7), we showed that methionine is oxidized to a significant extent.

Interestingly, the dose-dependence of the maximum concentration in methionine sulfoxide is not straightforward: the amount of methionine sulfoxide is larger for doses at 29 and 59 kGy than for doses at 106 and 260 kGy for bags aged less than six months (Figure 7b). Moreover, the oxidation level decreases with the ageing and is the same for all doses for ageing lasting more than six months.

## 4. Analysis at the Macromolecular Level

The changes monitored and evidenced during mechanical tests, permeation, and acidity are investigated at the macromolecular scale. That is, structural modificationsat surface, crystallinity, oxidation, thermal stability are investigated using different techniques such as FTIR, µ-ATR, XPS, DSC, combined with PCA and AComDim as chemometric techniques for analysis.

### 4.1. Crystallinity

**Figure 8:**
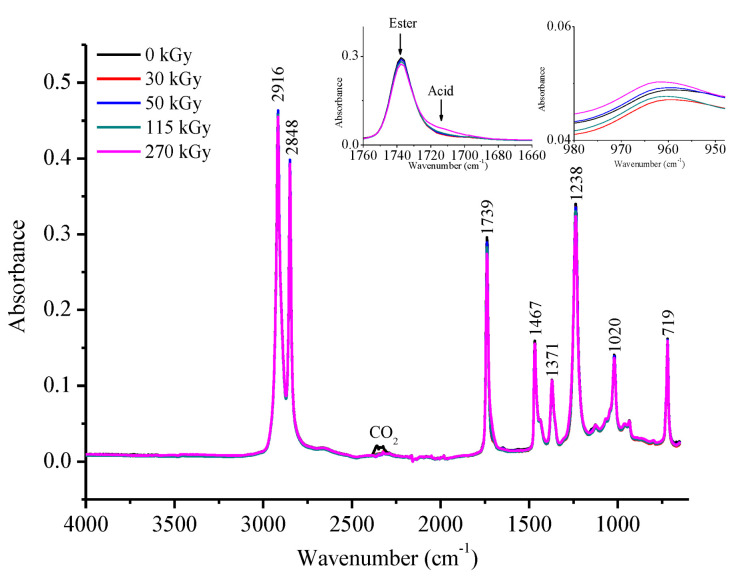
Overlay of EVA spectra for non-sterilized and γ-irradiated samples. Inset: enlargements of the carbonyl zone (**left**) and of the trans alkene zone (**right**). Reproduced from [28] with the permission of Polymer Degradation and Stability.

While it is possible to observe cross-linking events through the mechanical tests, FTIR analysis is not sensitive enough to detect their occurrence. The signals of the -CH_2_- and -CH_3_ groups are not modified, as highlighted in Figure 8: at 2916 and 2848 cm^−1^ for the asymmetric and symmetric stretching vibrations of the -CH_2_- group, respectively. The bands at 1474 and 730 cm^−1^ correspond to the crystalline phase and the bands at 1464 and 720 cm^−1^ correspond to the amorphous phase [28]. The intensity and location of these specific crystallinity bands [44,45,46,47,48] do not vary with respect to dose, therefore, the impact of γ-irradiation on the crystallinity of the EVA/EVOH/EVA film is not large enough to be detected by FTIR.

**Figure 9:**
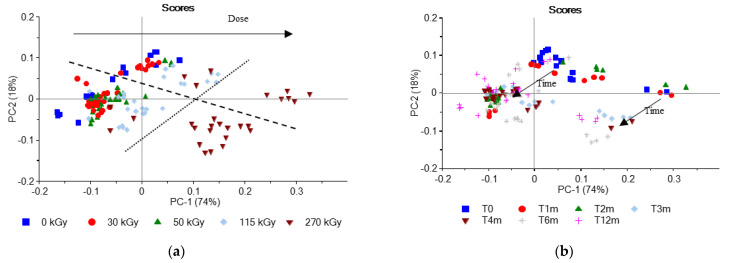

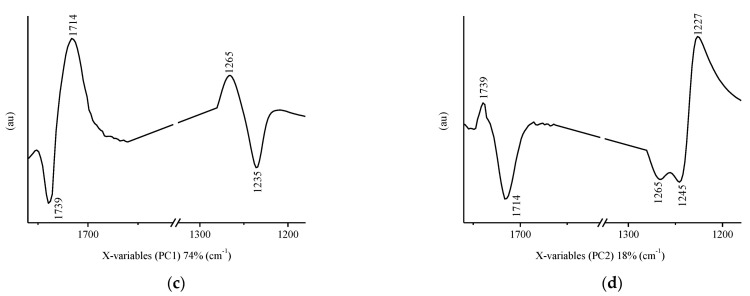
PCA of EVA spectra at all γ-irradiation doses (1760–1670 cm^−1^//1280–1180 cm^−1^). Spectra are baseline corrected and normalized. (**a**) Score plot of PCA with γ-dose labels. Dashed line delimits the ageing groups and dotted line delimits the γ-doses groups. (**b**) Score plot of PCA with time labels. (**c**) Loading plot of PC1. (**d**) Loading plot of PC2. Reproduced from [28] with the permission of Polymer Degradation and Stability.

FTIR analysis combined with PCA of the EVA film surface shows a peak at 1714 cm^−1^ corresponding to the formation of carboxylic acid and a peak at 1739 cm^−1^ corresponding to the ester group degradation for increasing γ-irradiation dose and over-ageing time [28], as highlighted in Figure 9. Hence, the effect of dose on the formation of carboxylic acid is only significant for doses larger than 50 kGy (Figure 9a,c), i.e., a significant increase in the amount of carboxylic acid, and the effect of ageing is only significant for ageing of more than two months (Figure 9b,d). These observations are correlated with the increase in acidity (Figure 4).

### 4.2. XPS Analysis

**Figure 10:**
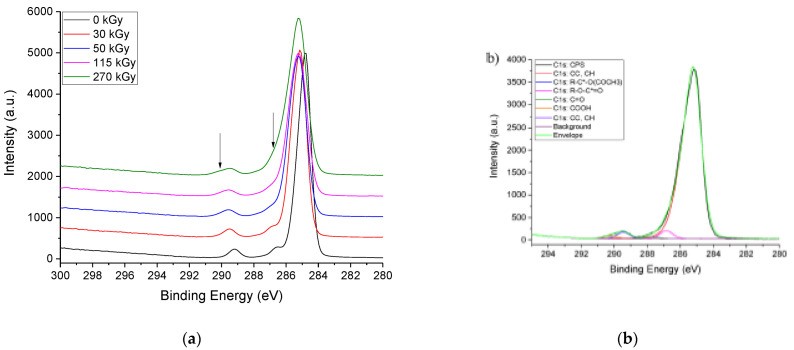
(**a**) C1s XPS spectra of EVA/EVOH/EVA film irradiated at different doses, (**b**) example of decomposition of the C1s spectrum recorded on sample irradiated at 270 kGy. Reproduced from [33] with the permission of Applied Surface Science.

The formation of the carbonylated compounds (carboxylic acids, ketones, aldehydes) and the degradation of the ester group (Figure 10a) when the γ-dose increases is also observed at the surface of the inner layer of EVA/EVOH/EVA film by XPS [33]. The degradation of ester fonctions is highligthed by the decrease of arrow tagged peaks in Figure 10a from non sterilizes film to 270 kGy-dose irradiated film (decomposition of XPS peak is displayed in Figure 10b). The generation of ketone and aldehyde is also highlighted with the XPS analysis. 

### 4.3. DSC Analysis

**Figure 11:**
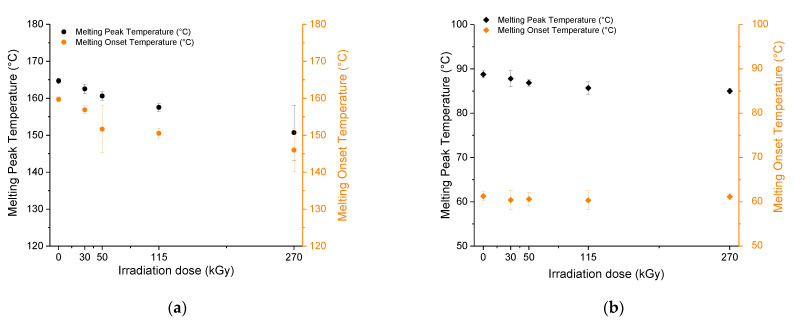
Melting peaks and melting onset points for the EVOH layer (**a**) and the EVA layer (**b**) used in the EVA/EVOH/EVA multilayer film according to the γ-irradiation dose. Reproduced from [34] with the permission of Journal of Applied Polymer Science.

The thermal analysis by DSC method [34] agrees with permeation experiments. Indeed, the clear changes (Δ*T* = 15 °C in Figure 11a) in temperatures of melting peak and of melting onsets with γ-irradiation doses highlight the degradation of EVOH layer at 270 kGy whereas the non significant changes (Δ*T* < 3 °C in Figure 11b) for the same parameters of the EVA layers denote a low level of modifications even up to 270 kGy.

### 4.4. FTIR AComDim

**Figure 12:**
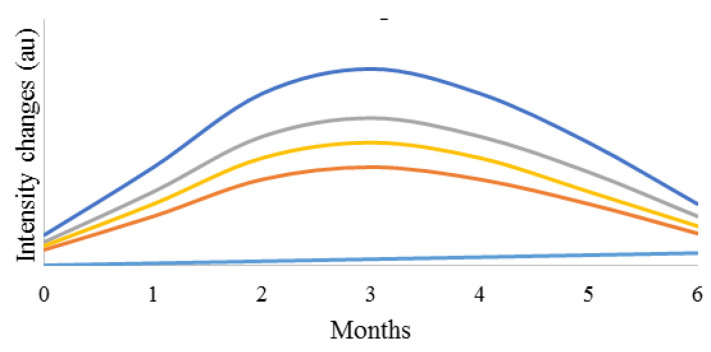
Schematic representation of the changes over time for the EVA materials. 0 kGy corresponds to the lowest curve and 270 kGy to the highest curve. Reproduced from [36] with the permission of Analytica Chimica Acta.

AComDim is applied to the FTIR spectra to highlight the main factors involved in the modification of the EVA/EVOH/EVA multi-layer film under γ-irradiation and over time [36]. This statistical method shows that the γ-dose, the natural ageing, and the γ-dose × natural ageing interaction are the most relevant factors. The chemical modifications that occur at the surface of the EVA/EVOH/EVA multi-layer film are proportional to the γ-irradiation doses. The kinetics observed over the ageing time reveals that the EVA multi-layer film after six months of ageing seems in its original state at t0 (i.e., no ageing). This kinetics is displayed with the schematic curves in Figure 12. Thus, the new molecules generated are probably volatile species.

## 5. Analysis at the Molecular Level

To get a deeper insight into the processes governing the changes in properties at both product and macromolecular levels, investigations of molecular processes and molecular species are performed using several techniques such as FT-IR, EPR, TOC, and HPLC titration.

### 5.1. EPR Analysis

γ-irradiation is one of the best techniques to generate radicals, as displayed in Scheme 1. Consequently, a part of the changes in properties must be related to the fate of these primary radicals generated by γ-irradiation. Therefore, one of the best techniques to detect and to monitor radical species is EPR. However, because of the experimental conditions it is not possible to detect any of the radicals or radical cations displayed in Scheme 1a and b. The hydroxyalkyl radical displayed in Scheme 1c is not detected for the EVA/EVOH/EVA film, while it could be observed for PE/EVOH/PE films for several weeks [29]. This difference is likely due to the thinner EVOH layer and the lesser VOH content in the EVA/EVOH/EVA film than in PE/EVOH/PE film. Nevertheless, we assume that the same radical species are generated in this EVOH layer in each film while different chemistries occur (vide infra).

### 5.2. Total Organic Compounds and Acidity

**Figure 13:**
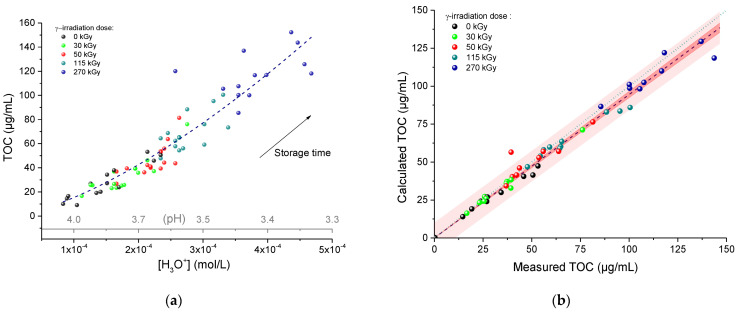
(**a**) TOC vs pH at different irradiation doses in EVA/EVOH/EVA films. Dotted line shows the trend. (**b**) Calculated TOC vs measured TOC at various doses of γ-irradiation in EVA/EVOH/EVA films. The light red band represents 95% prediction band and the dark red band represents 95 % confidence band. The dashed line shows the trend. The dotted line represents the bisector line. Reproduced from [32] with the permission of European Journal of Pharmaceutical Sciences.

Interestingly, product acidity provides valuable information at the molecular level when complementary analysis using titration is performed such as the determination of Total Organic Carbon (TOC), total carboxylic acid, and conductivity [32]. It is possible to determine TOC and to show that it increases with acidity (Figure 13a). TOC can be calculated assuming the generation of formic, acetic, and other C3-C6 acids, titrated by ionic chromatography, and compared with the experimental values (Figure 13b) and it highlights that formic and acetic acids are the main carboxylic acids generated under γ-irradiation.

**Scheme 5:**
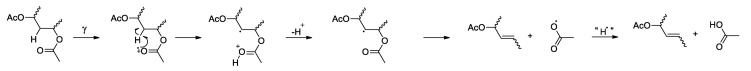
Radical pathway for the formation of acetic acid. Reproduced from [28] with the permission of Polymer Degradation and Stability.

The first acid production pathway (Scheme 5) is due to the γ-irradiation, which generates cation radicals on the carbonyl function. After H-abstraction, these cation radicals give alkyl radicals which promote the generation of carboxylic acids in a cascade of reactions.

### 5.3. HPLC Titration

**Figure 14:**
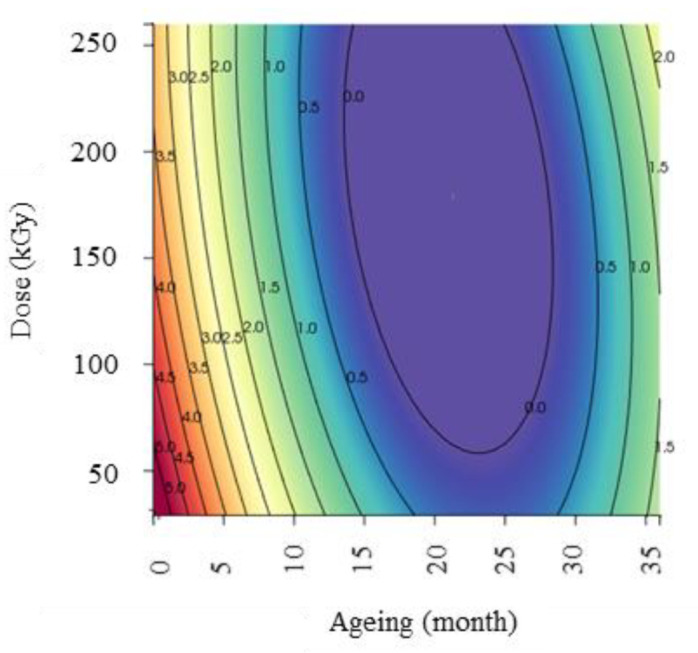
Response surface of the effect of ageing (months) vs dose (kGy) on the maximum methionine sulfoxide (contour in μM) concentration detected. Reproduced from [42] with the permission of Polymers.

As for the acidity, the oxidative power of a material is a product property whose determination affords valuable information on the presence of oxidants such as hydrogen peroxide, peroxides, and peracids. Unfortunately, these species cannot be differentiated from carbonyl and carboxylic functions by FT-IR and XPS. Therefore, HPLC titration is used to detect and monitor only the presence of oxidant species. As mentioned in the analysis at the product level section, methionine oxidation depends a lot on dose, ageing, and solution storage time [42]. Moreover, with a titration curve it is possible to determine three parameters, the maximal concentration of oxidant, the rate of oxidation, and the retardation time, useful to understand the efficiency of the oxidation process as a function of irradiation dose, ageing, and storage time. As an example, Figure 14 displays the change in maximal concentration of methionine sulfoxide (21 days of storage for the solution) depending on dose and ageing. Hence, almost no oxidation is observed after 20 months of ageing whatever the dose. On the other hand, maximum oxidation is observed for ageing shorter than five months and doses lower than 150 kGy. This counter-intuitive result is due to the degradation of peracids/peroxides/hydrogen peroxide when high doses are applied. Furthermore, the lower oxidation efficiency with ageing is assumed to be due to the evaporation of acetic acid with time.

## 6. The Chemistry behind γ-Irradiation of EVA/EVOH/EVA Multi-Layer Film

**Scheme 6:**
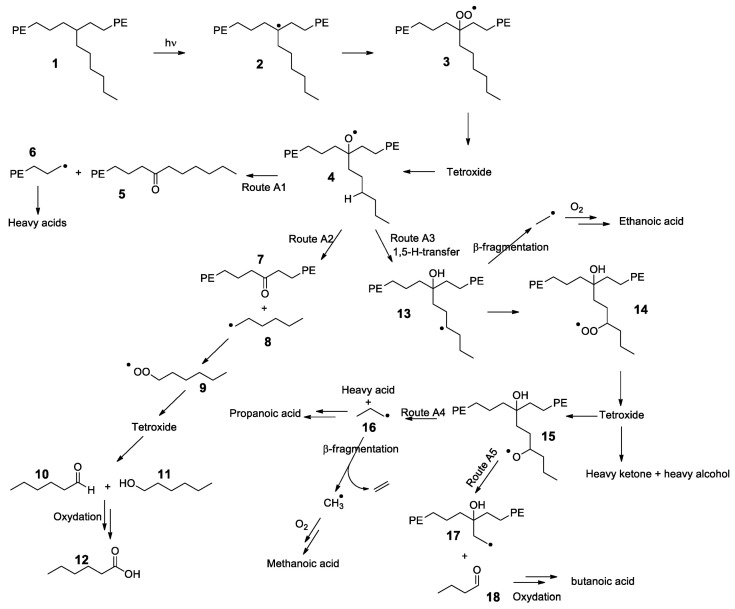
Degradation of PE in the presence of O_2_. Reproduced from [32] with the permission of European Journal of Pharmaceutical Sciences.

The radicals directly generated from the γ-irradiation (Scheme 1) cannot account for the reactivity observed at the product level. Importantly, the oxidation of methionine points at the compulsory presence of hydrogen peroxide/peroxides/peracids generated from the degradation of the EVA and EVOH layers. Therefore, compiling all observations done at each level helped us to propose mechanisms involving radical species leading to end-products accounting for the change in properties observed. Noteworthily, the PE co-polymer in EVA and EVOH plays a minor role in the species generation. Nevertheless, the reactivity of the radicals generated in the PE chain (Scheme 1a) is assumed to be the same as the reactivity observed for pure PE (Scheme 6). Hence, other carboxylic acids than formic and acetic acids are products of the degradation of PE co-polymer chains in EVA and EVOH.

**Scheme 7:**
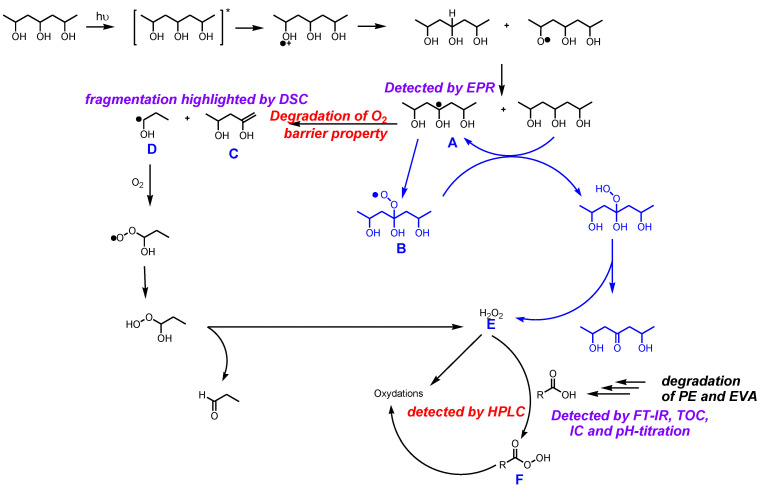
Generation of hydrogen peroxide upon γ-irradiation of polyvinyl alcohol polymer block of EVOH.

The γ-irradiation of an EVOH chain affords mainly hydroxyalkyl radicals **A** (Scheme 7). Then, these radicals decay via either fragmentation and further oxidation reactions or scavenging by O_2_ to afford peroxyl radicals **B** (blue route in Scheme 7). The latter react with another EVOH chain to regenerate hydroxyalkyl radicals **A** and to yield hemiacetal hydroperoxides, which collapse into ketones and hydrogen peroxide needed for the oxidation of biological solutions. However, this pathway is expected to be of low occurrence as radicals **A** are not detected by EPR in the EVA/EVOH/EVA film. On the other hand, the fragmentation of **A** affords a material of lower quality (poorer thermal properties detected by DSC) and hydroxyalkyl radical **D,** which reacts with O_2_ and H-donor to afford a hemiacetal peroxide, which collapses into aldehyde and hydrogen peroxide. The latter can both react with acetic acid generated from the degradation of the EVA layers to give peracetic acid for the oxidation of methionine and directly oxidize methionine. Retardation period, slow oxidation kinetics, and dependence on ageing point at the occurrence of the formation of peracetic acids and to the non-significant role in the modifications of the inner surface.

Other decomposition pathways are not described here because they afford only different types of alcohols, aldehydes, ketones, and carboxylic acids of various mobilities.

**Scheme 8:**
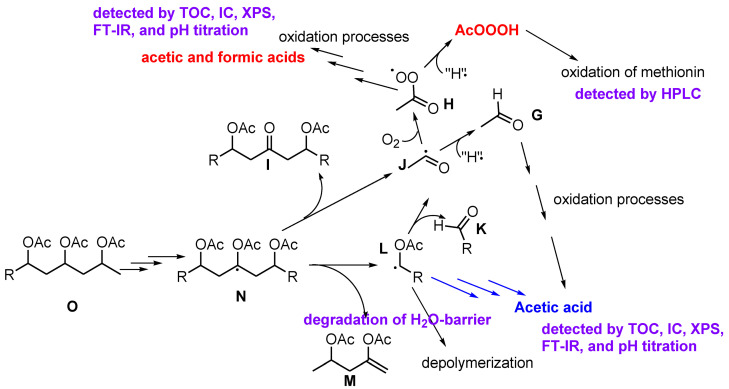
Degradation of EVA layers under g-irradiation and the subsequent manifolds.

Thus, γ-irradiation of polyvinyl acetate block **O** affords alkyl radical **N** (Scheme 1b), which spontaneously collapses either in ketone **I** and acyl radical **J** or in enolacetate **M** and alkyl radical **L** (Scheme 8). The latter decomposes through several manifolds: (i) depolymerization leading to chain length shortening, (ii) electron loss and a series of hydrolysis reactions (blue manifold) to afford acetic acid, as reported above; (iii) fragmentation to afford acyl radical **J** and aldehyde **K**. The impact of manifold (i) is easily detected through the changes in water permeation (vide supra) although DSC does not report significant alteration of the materials, as confirmed by tiny changes in the FT-IR signal. The impact of manifold (ii) is observed in the pH changes as well as in TOC and IC analyses. In manifold (iii), acyl radical **J** reacts via either H-abstraction, to yield acetaldehyde **G,** which is further oxidized into acetic acid, or cross-coupling reaction with O_2_ to afford acetyl peroxyl radical **H**. The latter reacts via either further oxidation processes to yield acetic and formic acids or H-abstraction to yield peracetic acid needed for the oxidation of methionine.

## 7. Conclusions

This work highlights nicely the interest of the multi-level and multi-technique approach as striking changes in pH, acidic species, permeation, and oxidation are observed whereas mechanical and physical features are not significantly alterated for the EVA layers. Thus, this multi-level approach provided us with a deep understanding of the mechanism leading to striking changes in some properties.

**Figure 15:**
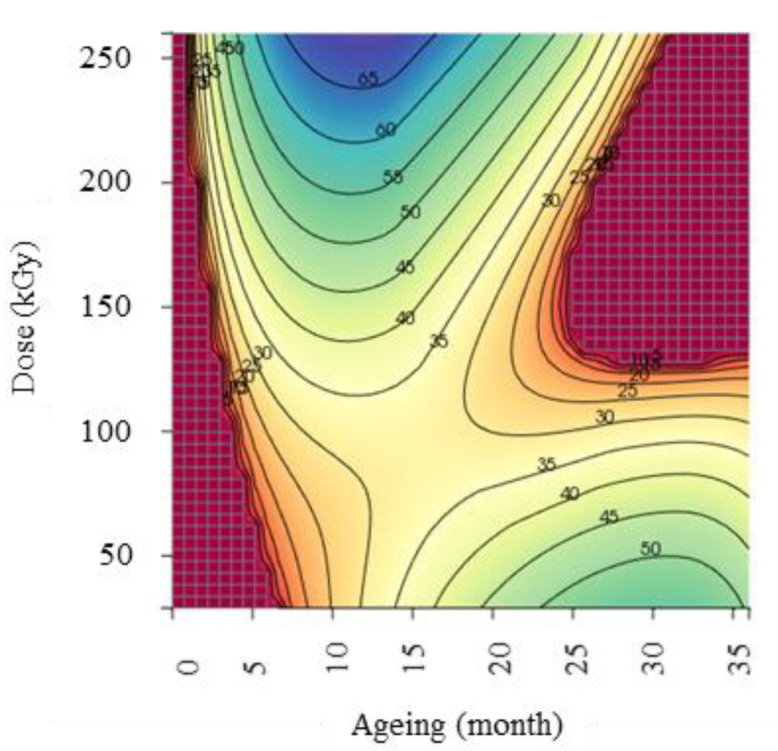
Desirability plots of ageing (months) vs dose (kGy). Contours are in % of desirability. Deep blue colour is for 100% of desirability (all requirements are fulfilled) and red colour is for 0% of desirability (no requirements fulfilled). Reproduced from [42] with the permission of Polymers.

Importantly, our approach allowed us to develop a multifactorial analysis using chemometrics leading us to apply a full factorial design model. For example, in the case of the oxidation process, which plays a crucial role in the solution stability, the full factorial design approach showed us how dependent the oxidation process is on ageing, storage time, and dose. Hence, the perfect conditions for suppressing oxidation issues would be: oxidation induction time of more than 10 days, oxidation rate close to or less than 3 mM.day^−1^, and level of oxidation lower than 3 mM. Then, it is possible to define a desirability function that provides a response surface as displayed in Figure 15 [42]. Hence, for 50 kGy, the maximum of desirability (50%) is reached for long ageing (30 months).

**Figure 16:**
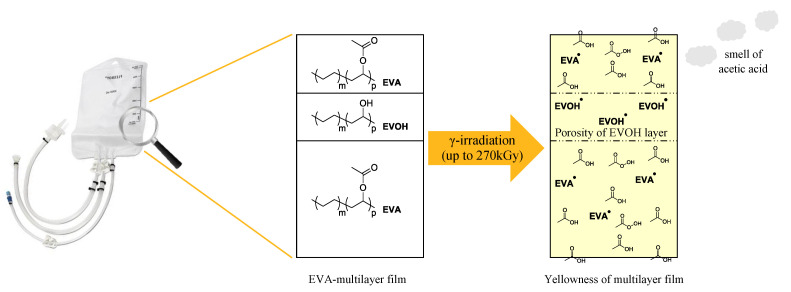
Changes induced in the EVA multilayer film by γ-irradiation.

Importantly, except for the acidity, permeation and oxidation properties, the investigated properties and the material integrity are not significantly altered for the irradiation doses below 50 kGy that are usually applied in industrial sterilization. Thus, the chemical changes observed after γ-irradiation of the EVA multi-layer film are summarized in Figure 16.

The powerfulness of this approach as well as its impact on results provided to end-users led us to envision the set-up of an international consortium, team Nablo, gathering teams from Pacific Northwest National Laboratory (Richland, WA, USA), Texas A&M University (College Station, TX, USA), and Aix-Marseille University (Marseille, France) as well as several companies such as Sartorius. This consortium encompasses all fundamental aspects: physics, chemistry, mechanics, and mathematics, from irradiation aspects to applications by end-users in biopharmaceutical and medical fields to assess likewise the impact of alternative irradiation modalities such as X-ray and E-beam to supplement the sterilization by γ-rays [49].

## Data Availability

Not applicable.
